# Understanding mitochondrial myopathies: a review

**DOI:** 10.7717/peerj.4790

**Published:** 2018-05-21

**Authors:** Abhimanyu S. Ahuja

**Affiliations:** Wilkes Honors College, Florida Atlantic University, Jupiter, FL, United States of America

**Keywords:** Genetic mutations, Pathology, Treatment

## Abstract

Mitochondria are small, energy-producing structures vital to the energy needs of the body. Genetic mutations cause mitochondria to fail to produce the energy needed by cells and organs which can cause severe disease and death. These genetic mutations are likely to be in the mitochondrial DNA (mtDNA), or possibly in the nuclear DNA (nDNA). The goal of this review is to assess the current understanding of mitochondrial diseases. This review focuses on the pathology, causes, risk factors, symptoms, prevalence data, symptomatic treatments, and new research aimed at possible preventions and/or treatments of mitochondrial diseases. Mitochondrial myopathies are mitochondrial diseases that cause prominent muscular symptoms such as muscle weakness and usually present with a multitude of symptoms and can affect virtually all organ systems. There is no cure for these diseases as of today. Treatment is generally supportive and emphasizes symptom management. Mitochondrial diseases occur infrequently and hence research funding levels tend to be low in comparison with more common diseases. On the positive side, quite a few genetic defects responsible for mitochondrial diseases have been identified, which are in turn being used to investigate potential treatments. Speech therapy, physical therapy, and respiratory therapy have been used in mitochondrial diseases with variable results. These therapies are not curative and at best help with maintaining a patient’s current abilities to move and function.

## Introduction

Mitochondria are small organelles within cells that generate energy for cells. They can be considered to be a cell’s “power-plant” (National Institute of Neurological Disorders and Stroke (NINDS), 2018). Approximately 90 percent of the energy needs of the body’s tissues are met by mitochondria (National Institute of Neurological Disorders and Stroke (NINDS), 2018). This energy is stored in a molecule known as Adenosine Triphosphate (ATP). ATP is the product of a long chain of biochemical and biophysical events that convert sugar and oxygen into energy. The ATP molecule is needed for cellular metabolism and is transported out of the mitochondria into the main part of the cell (Muscular Dystrophy Association (MDA), 2009c).

### Pathology of mitochondrial myopathy

When either the Mitochondrial DNA (mtDNA) or Nuclear DNA (nDNA) is abnormal, the mitochondria cannot produce the energy needed by cells for their metabolism and diseases occur. We can think of this as a power failure within the body. The impact of such power failure within the body can range from mild to severe and even can lead to death. Genetic mutations in either mtDNA or nDNA coding for mitochondria are responsible for disease (Muscular Dystrophy Association (MDA), 2009c).

nDNA defects tend to be autosomal recessive and are transmitted in a Mendelian fashion where half of the genetic material of a fertilized egg derives from each parent. Since the egg has multiple mitochondria while sperm has virtually none, mutations of mitochondrial genes in mtDNA are transmitted through the maternal line, or they may be sporadic (Agamanolis, 2017). The number of mitochondria within cells varies depending on the energy needs of the cells. Cells which have more ATP requirements will have more mitochondria. As an example, muscle cells and heart muscle cells need more energy than the cells of the liver and have more mitochondria to generate more ATP. The heart muscle cells have about 5,000 mitochondria and each liver cell contains 1,000–2,000 mitochondria (Alberts et al., 2002). Cells are unevenly affected by abnormal mtDNA. This is because a cell may have some mitochondria containing normal (referred to as wild type) mtDNA while the other mitochondria within the same cell may contain mutant mtDNA.

Mitochondria segregate in a random way when cells divide and so the percentage of wild type and mutant mtDNA coexisting in any given cell is variable and this is known as heteroplasmy (Agamanolis, 2017). After several cell division cycles, a cell may contain mitochondria with a majority of mutant mtDNA and when the proportion of such mutant mtDNA in mitochondria exceed a threshold of 80–90%, cellular disease becomes manifested. Due to heteroplasmy, mitochondrial disease takes many forms and even family members may present differently with the disease.

Diseased muscle fibers containing groups of abnormal mitochondria look to be “Ragged Red Fibers” when the muscle is tinged with Gomori trichome stain. These red ragged fibers are specific to mitochondrial disorders but are present in only 1/3rd of these disorders (Agamanolis, 2017). A mitochondrial disease causing prominent muscular problems is referred to as a mitochondrial myopathy. Due to their high energy requirements, organs such as the brain and skeletal and cardiac muscles are disproportionately affected by mitochondrial dysfunction and are collectively referred to as mitochondrial encephalomyopathies.

### Review methodology

Scholarly articles that were reviewed in this paper were searched in journal databases and subject-specific professional websites. The search terms that were used when searching for articles included mitochondrial myopathy, genetic mutations, and treatment. Inclusion criteria for selected articles required that articles be directly related to the topic on mitochondrial myopathy and be peer reviewed. Both qualitative and quantitative articles were reviewed. Qualitative articles provide insights into the problem by helping understand reasons, opinions, and motivations. Quantitative articles on the other hand use measurable data to formulate facts and discover patterns in research.

## Causes, Risk Factors, Symptoms

### Causes

Mitochondrial diseases are caused by mutations in mtDNA or nDNA. These mutations can be inherited or spontaneously engendered, which alters the function of the proteins or RNA molecules that are usually a part of mitochondria (United Mitochondrial Disease Foundation, 2017a). Mitochondrial diseases such as Leigh Syndrome and Mitochondrial Neurogastrointestinal Encephalopathy Syndrome (MNGIE) that are caused by nDNA mutations and even mitochondrial DNA depletion syndrome (MDS) are autosomal recessive, requiring mutations in copies of a gene from both parents to induce disease (Muscular Dystrophy Association (MDA), 2009a). So, essentially the person must be homozygous recessive, i.e., have two copies of the recessive allele for manifestation of a mitochondrial disease. For example, more than one defect may cause Leigh’s Syndrome. Such defects include a pyruvate dehydrogenase (PDHC) deficiency, and respiratory chain enzyme defects—complexes I, II, IV, and V (Muscular Dystrophy Association (MDA), 2009a). The mode of inheritance can vary based on the type of defect. It may be X-linked dominant with the defect on the X chromosome and disease usually occurs in males only. It may also be autosomal recessive or maternal. Some cases may be spontaneous and these are not inherited.

mtDNA is inherited maternally. This is because only the mother’s egg cell contributes mitochondria to an embryo and the father’s sperm does not do so (Muscular Dystrophy Association (MDA), 2009a). It should however, be noted that not all mitochondrial mutations are inherited. Some of the mutations can occur during embryonic development in the womb (Muscular Dystrophy Association (MDA), 2009a).

Considering that mitochondrial dysfunction may originate from mutations in more than 1,000 genes, the type of mutation may vary greatly. Scarpelli et al. (2018) report two mtDNA mutations in mitochondrial myopathy patients. Specifically, the paper identifies the m.8305C>T mutation in the MTTK gene and the MTTM gene m.4440G>A mutation (Scarpelli et al., 2018). The authors conclude that mutations in mitochondrial tRNAs represent hot spots for mitochondrial myopathies in adults. Another recent paper by Bartsakoulia et al. (2018) identifies mutations in MIEF2 gene that results in imbalanced mitochondrial dynamics and a combined respiratory chain enzyme defect in skeletal muscle, leading to mitochondrial myopathy.

There are too many variants of mitochondrial myopathies; as such it must be stated that analysis of each specific type is beyond the scope of this paper. For example, some diseases such as Myoclonic Epilepsy and Ragged-Red Fiber (MERRF) are caused by point mutations in the mitochondrial DNA, while Pearson Syndrome is usually sporadically inherited and is caused by large deletions of mtDNA. Such deletions can range from 1,000 to 10,000 DNA building blocks (nucleotides). The mtDNA deletions involved in Pearson marrow-pancreas syndrome impair oxidative phosphorylation and decrease the energy available to cells.

Genes within the mitochondria normally code for proteins that work inside the mitochondria. Proteins within each mitochondrion manufacture ATP in an assembly line fashion. They consume fuel molecules in the form of sugar and fat, in the presence of oxygen to manufacture ATP (Muscular Dystrophy Association (MDA), 2018a). Within the mitochondrial matrix, the Krebs cycle, or the citric acid cycle, generates the high-energy electron carriers NADH and FADH_2_. These are generated through the oxidation of acetyl CoA to CO_2_ coupled with the reduction of NAD+ and FAD. Five protein complexes referred to as complexes I, II, III, IV and V are embedded in a tightly folded membrane called the inner mitochondrial membrane. During oxidative phosphorylation, the protein complexes carry out chemical reactions that drive the production of ATP. An unequal electrical charge is created on either side of the inner mitochondrial membrane through a step-by-step transfer of electrons (released from NADH and FADH_2_) coupled with movement of protons into the intermembrane space which creates an electrochemical gradient across the inner membrane. Complexes I through IV, which constitute the electron transport chain, move the electrons down the assembly line. Complex V which actually makes the ATP is called ATP synthase (Muscular Dystrophy Association (MDA), 2018a). Protons flow back into the matrix down their concentration gradient through ATP synthase resulting in the synthesis of ATP.

Defects in one or more these complexes typically cause mitochondrial disease (Muscular Dystrophy Association (MDA), 2018a). Muscle and nerve cells have high energy requirements which need ATP generated by mitochondria and hence, are very sensitive to mitochondrial defects. Besides not being able to produce ATP, mitochondrial defects can lead to an excess formation of harmful oxygen radicals within cells (Muscular Dystrophy Association (MDA), 2018a).

### Risk factors

Risks of inheriting mitochondrial disease vary based on whether the mutations are in nDNA or mtDNA. For an autosomal recessive disorder it takes two mutated genes in nDNA to cause illness (Muscular Dystrophy Association (MDA), 2009a). A person with one gene mutation for an illness that requires two mutations to produce the illness is said to be a carrier (Muscular Dystrophy Association (MDA), 2018a). Assuming both parents are carriers for an autosomal recessive disorder, the likelihood of a child inheriting this disorder is 25 percent with each conception (Muscular Dystrophy Association (MDA), 2018a).

With respect to mitochondrial disorders caused by mutations in mtDNA the offspring of heteroplasmic mothers with mitochondrial DNA mutations are at risk of developing the same disorder. The mother’s egg cells contain both normal (wild type) and mutant mitochondria. Some egg cells may have just a few mutant mitochondria and so an offspring from one of these cells is likely to be healthy but an egg cell with more mutant mitochondria is likely to cause the disease in the offspring (Muscular Dystrophy Association (MDA), 2009a). The severity of the offspring’s disorder is dependent on how many normal versus mutant mitochondria the offspring inherits. Therefore, the risk of inheriting disease through mtDNA is variable. Finally, mitochondrial disease can also occur with no family history in a sporadic manner (Muscular Dystrophy Association (MDA), 2009a). Special care must be taken during pregnancy as this disorder can also occur at the embryonic stage. Another risk factor is a family history of the disease.

A person with a mitochondrial disease is at special risk immediately following an illness including colds, flu, and infections which can cause degradation in the person’s overall health condition and possibly even lead to death. Any illness needs prompt medical treatment.

### Symptoms

Muscle weakness, muscle atrophy, and exercise intolerance which refer to exhaustion caused by physical exertion are some of the main symptoms of mitochondrial myopathy (Muscular Dystrophy Association (MDA), 2018b). These symptoms can vary even within the same family. Often times, weakness begins with the muscles of the eyes and the eyelids. This leads to progressive external ophthalmoplegia (PEO) or the progressive paralysis of eye movements, and ptosis which can be one of the first symptoms of this disease (Muscular Dystrophy Association (MDA), 2018b). Some of these symptoms may not be apparent till the patient is older.

Mitochondrial myopathies often cause weakness with other facial and neck muscles. This can cause speech to become slurred and more importantly, swallowing can become difficult or even impossible which can lead to aspiration into the lungs (Muscular Dystrophy Association (MDA), 2018b). People with mitochondrial myopathies experience increasing weakness in arms and/or legs and may eventually be unable to walk and/or use their arms (Muscular Dystrophy Association (MDA), 2018b). Another life-threatening symptom is weakness in the muscles that support breathing and consequently needing ventilator support to breathe (Muscular Dystrophy Association (MDA), 2018b).

Symptoms of mitochondrial encephalomyopathy include the above symptoms of mitochondrial myopathy in addition to hearing impairment, and migraine-like headaches and seizures often accompanied by stroke-like episodes (Muscular Dystrophy Association (MDA), 2018b). Vision can also be affected due to optic atrophy (shrinkage of the optic nerve) or retinopathy which affects the retinal cells at the back of the eye (Muscular Dystrophy Association (MDA), 2018b). Ataxia (difficulty with coordination and balance) can also occur, leading to falls (Muscular Dystrophy Association (MDA), 2018b).

Mitochondrial disease can also cause cardiac problems including conduction block (problem with rhythmic heart beats) and damage to cardiac muscle, kidney disease, gastrointestinal disorders, and/or diabetes (Muscular Dystrophy Association (MDA), 2018b).

There are specific pediatric concerns. Muscle weakness and/or brain abnormalities in children can lead to developmental delays (Muscular Dystrophy Association (MDA), 2018b). They may take longer to sit, crawl and walk (Muscular Dystrophy Association (MDA), 2018b). They may also have speech difficulties and other learning disabilities (Muscular Dystrophy Association (MDA), 2018b). So called “red flags” for mitochondrial disease in children include short stature, neurosensory hearing loss, ophthalmoplegia, ptosis, neuropathy or nerve disease, diabetes, hypertrophic cardiomyopathy or enlarged cardiac muscle, and renal tubular acidosis (Koenig, 2008).

## Prevalence Statistics

Mitochondrial diseases are more common than was earlier believed. As many as 2 million Americans are afflicted by mitochondrial disease (Lemonick, 2006). According to the United Mitochondrial Disease Foundation (UMDF), “every 30 min a child is born who will develop a mitochondrial disease by age 10”. In the United States the incidence of mitochondrial disease is about one in 4,000 individuals. This is roughly the prevalence of cystic fibrosis of Caucasian births in the US (MitoAction). According to estimates from the Mitochondrial and Metabolic Disease Center at UC San Diego there are 1,000–4,000 births each year in the US with mitochondrial disorders (Powledge, 2014).

The exact incidence of mitochondrial disease is hard to know. Global statistics have varied substantially over time. According to the Australian Mitochondrial Disease Foundation (AMDF), about one in 250 people are carriers of mitochondrial genetic defects which is roughly equal to about 90,000 Australians. Many of these people will not develop mitochondrial disease and may instead develop mild symptoms that may not be noticeable during their lifespan. According to AMDF estimates, the likelihood of developing significant mitochondrial disease in the overall population is about one in 5,000 (Australian Mitochondrial Disease Foundation (AMDF), 2017).

According to the most recent study published in January 2015 by the Wellcome Trust Centre for Mitochondrial Research at Newcastle University about 2,473 women in the UK and 12,423 women in the US, aged between 15 and 44 years, carry mutations in their mitochondrial DNA and can potentially pass mitochondrial disease to their children (Science 2.0, 2015). This is an average of 152 births per year in the UK and 778 births per year in the US (Science 2.0, 2015). This study excluded the impact of ethnic diversity and variations in fertility rates within the UK.

The prevalence figures based on patients’ already clinically diagnosed may actually be on the low side and represent conservative minimum estimates (Martikainen, 2012). This could be because identified patients may be those with most severe clinical symptoms. Patients with less severe symptoms may go unnoticed and this hinders identification of those patients who do not fit the stereotype of the ‘typical’ patient. Furthermore, prevalence data for mitochondrial diseases are available only for few populations such as in the US and parts of Europe. Similar data for countries in Asia and elsewhere is less available. Prevalence figures can vary substantially between different population groups for many diseases. In mitochondrial diseases too, there seems to be variation between populations in the prevalence of mitochondrial disease (Martikainen, 2012).

## Treatments

We have no cure for mitochondrial disease as of today. Treatment remains supportive for the most part and emphasizes symptom management. However, some of the potential manifestations of mitochondrial diseases are prevalent in the overall population. Hence complications such as heart problems, stroke, seizures, migraine, and diabetes can be treated with medications, dietary changes, and lifestyle changes (Muscular Dystrophy Association(MDA), 2009b). Unfortunately, due to the often progressive nature of this disease, more and more symptoms and complications arise in many patients leading to significant morbidity and mortality.

### Pharmaceutical treatments

More recent and not fully proven treatments seek to fix or bypass defective mitochondria. These treatments are not strictly drugs but rather supplements based on three natural occurring substances involved in ATP generation in cells (Muscular Dystrophy Association (MDA), 2009b). As such, they do not have the major side effects of pharmaceutical drugs though they can cause some side effects described below. These supplements are not efficacious in all people, though some people are helped by them.

The first substance, creatine, is a reserve for ATP and forms a compound called creatine phosphate (Muscular Dystrophy Association (MDA), 2009b). Creatine releases phosphate when a cell’s mitochondria cannot meet the cell’s demand for ATP. This is how the initial burst of ATP needed for demanding muscle activity is generated. However, this type of energy burst is short-lived. The standard dosage for adults is 10 grams/day, split into two doses. The pediatric dosage is 0.1 gram/kg per day, split into two doses (Parikh et al., 2009). The main side effect is gastrointestinal upset in some cases (Parikh et al., 2009).

Another substance, carnitine, helps improve the efficacy of ATP production by helping pull in specific fuel molecules such as fatty acids into mitochondria (Muscular Dystrophy Association (MDA), 2009b). Fatty acids are used as an energy substrate in all tissues except the brain. Carnitine is known as L-Carnitine supplement over-the-counter. The standard oral dose in children is 20–100 mg/kg/day split into two or three doses. The usual adult dose is 330–990 mg/dose twice or thrice per day, depending on clinical response (Parikh et al., 2009). One side effect is body odor caused by bacterial breakdown of carnitine. This can be treated by dose reduction. Gastrointestinal upset is another side effect (Parikh et al., 2009).

Lastly, coenzyme Q10, or coQ10, is a treatment target. It is a component of the electron transport chain that uses oxygen to create ATP (Muscular Dystrophy Association (MDA), 2009b). In some cases mitochondrial diseases are due to by coQ10 deficiency. In such cases supplementation with coQ10 may be of help. Recently CoQ10 is available in the form of ubiquinol with better absorption by the body. Standard dose of ubiquinol is 2 to 8 mg/kg per day twice a day with food (Parikh et al., 2009). Coenzyme Q10 is usually not prescribed for children. The main side effect is wakefulness (Parikh et al., 2009).

Creatine, L-carnitine and coQ10 supplements have been combined into a “mito cocktail” for treating mitochondrial disease. Although they do not work in many cases, they do provide modest benefits to some patients. The clinical ability to achieve optimal dosing of medications is limited and the long-term benefits of treatment are unknown at this time.

## Prevention

Mitochondrial diseases can have severe consequences, particularly in tissues with high energy requirements such as the brain, muscle (including heart), liver and kidney. Genetic counseling of couples, family history analysis, and prenatal diagnosis are important.

New In-Vitro-Fertilization (IVF) based techniques have been developed in the UK which may help in the prevention of maternally transmitted mitochondrial disease. The technique is called ‘mitochondrial donation’ and involves the removal of defective mitochondria inherited from the maternal line and replacing them with healthy mitochondria from another woman. The nuclear DNA, which contains 99.9% of genetic material from both parents, remains the same (Science 2.0, 2015).

Researchers at the Wellcome Trust Centre for Mitochondrial Research at Newcastle University will be the first to offer mitochondrial donation if the UK parliament agrees to new regulations of the Human Fertilization and Embryology Act (1990) (Science 2.0, 2015). Consequently this approach towards prevention of mitochondrial disease can have significant positive impact in those countries that are considering permitting the donation of mitochondrial.

## Research and Clinical Trials

Muscular Dystrophy Association (MDA) funded scientists have identified several of the genetic defects that cause mitochondrial diseases (Muscular Dystrophy Association (MDA), 2009d). Based on this information related to genetic defects animal models of mitochondrial disease can be created. These can in turn be used to research new approaches to treating mitochondrial diseases. Some researchers are researching mechanisms to add therapeutic genes to mitochondria (Muscular Dystrophy Association (MDA), 2009d). Still others are researching biochemical processes within mitochondria that can either correct or help treat mitochondria defects. This may or may not involve the addition of new genes.

Recent research has shown, rather unexpectedly, that mitochondria can cross cell boundaries and thus be horizontally transferred between cells (Berridge et al., 2016; Torralba, Baixauli & Sánchez-Madrid, 2016). Based on the *in vitro* and *in vivo* results, Berridge et al. (2016) conclude that there is a high likelihood that mitochondria not only can, but do move between cells under normal physiological conditions such as during development and maintenance of tissue homeostasis. According to Torralba, Baixauli & Sánchez-Madrid (2016), the possible exchange of healthy mitochondria to sustain cells with oxidative phosphorylation defects could prevent the manifestation of some diseases associated with mitochondrial dysfunction. Additional research is required to assess the actual impact of these mechanisms in mitochondrial diseases.

Mancuso et al. identify a set of recommended outcome measures to be implemented in mitochondrial myopathy clinical studies (Mancuso et al., 2017). Patient-reported quality of life, fatigue, and pain questionnaires were identified as being important (Mancuso et al., 2017). The authors propose a set of clinical scales, functional tests, performance and patient-reported outcome measures, and biomarkers to be applied to both adults and children affected by mitochondrial myopathy (Mancuso et al., 2017). According to Nightingale et al. (2016), mitochondrial defects caused by nuclear genes offer the greatest potential when seen from a clinical perspective. Pre-clinical studies have identified bezafibrate as a disease-modifying pharmaceutical agent that helps improve mitochondrial function using both cellular and animal models of mitochondrial myopathy (Noe et al., 2013; Komen & Thorburn, 2014). Clinical evaluation of bezafibrate to stimulate mitochondrial biogenesis is in progress (Nightingale et al., 2016; Clarke et al., 2016).

EPI-743 is a drug that is currently in clinical trials in the United States and Europe. EPI-743 was recently granted orphan drug designation by the FDA to treat patients who are seriously ill and have inherited mitochondrial respiratory chain disorders (United Mitochondrial Disease Foundation, 2013). EPI-743 helps to regulate cellular energy metabolism by targeting an enzyme NADPH quinone oxidoreductase 1 (NQO1). EPI-743 was discovered and developed by Edison Pharmaceuticals. Patients with genetically confirmed mitochondrial disease, and patients without genetic confirmation who meet specific clinical criteria, are both eligible for these clinical trials (United Mitochondrial Disease Foundation, 2013). So far 40 patients have been treated for over 7,000 days and there have been no significant drug-related side effects (United Mitochondrial Disease Foundation, 2013). The clinical trial is designed to be 13 weeks long. It is clinically indicated in Leigh’s Syndrome and Leber’s Hereditary Optic Neuropathy (LHON) (United Mitochondrial Disease Foundation, 2013).

## Therapeutic Approaches

Therapeutic approaches such as physical therapy, speech therapy or respiratory therapy may be required in some mitochondrial disease patients (United Mitochondrial Disease Foundation, 2017b). They tend to be supportive in nature and while these therapies will not reverse the disease process, they may help to retain or even improve the patient’s present levels of mobility, functioning, and strength (United Mitochondrial Disease Foundation, 2017b). There is no sure way of stopping mitochondrial disease from advancing and the ability to forecast therapeutic effectiveness in individual patients at the clinical level remains limited.

Physical Therapy: Mitochondrial patients can have limited functionality and strength. Their stamina and endurance can also be limited. Physical therapy can assist in maintaining muscle function and preventing joint contractures (El-Hattab & Scaglia, 2013). As an example, in Mitochondrial Depletion Syndrome (MDS) physical therapy has been shown to aid in the maintenance of muscle function (El-Hattab & Scaglia, 2013). A physical therapist (PT) needs to conduct a preliminary evaluation to develop a patient-specific treatment plan to help improve balance, gait, or endurance as needed. Therapy should begin at very low intensity for short time periods and should progress slowly from the patient perspective (Millhouse-Flourie, 2016b). PT’s can fine-tune the program so that when the patient feels well on a specific day the program can go forward, but on a ”bad day” exercise can be restricted to postural exercises or very slow movements.

Speech Therapy: Speech therapists help in the treatment and prevention of speech, cognitive, voice, communication, and swallowing disorders (Millhouse-Flourie, 2016b). They design exercise and other interventions to help strengthen speech and respiratory musculature (Millhouse-Flourie, 2016a). Treatment of swallowing difficulty or dysphagia includes strengthening exercises, safety strategies, and diet modifications to reduce the effects of incorrect swallowing which include malnutrition, dehydration, and respiratory problems such as aspiration pneumonia (Millhouse-Flourie, 2016a). As an example, in children with MELAS syndrome symptomatic treatment can help in facilitating swallowing (Vandana et al., 2015).

Respiratory Therapy: Mitochondrial disease can affect the lungs causing respiratory weakness or failure (Millhouse-Flourie, 2016a). Respiratory therapists recommend approaches to improve ventilation and assist attempts to cough and bring up secretions. Treatment can include occasional oxygen supplementation to permanent support on a ventilator. A case report shows for example in a patient with Leigh syndrome that ventilator support can be necessary (Shrikhande, 2010). Respiratory therapy is critical for the management of the patient with respiratory difficulties if they need to have anesthesia and surgery (Millhouse-Flourie, 2016a).

Diet: Dietary needs are extremely variable from patient to patient with mitochondrial myopathies. It is required to evaluate a patient’s nutritional deficiencies. Potential therapies include supplementing caloric intake, enteral feeding, increasing the frequency of meals, limited fasting, and parenteral nutrition (Parikh et al., 2009).

Treatment of manifestations: While treatment is generally supportive and includes physical and occupational therapy, care has to be taken to address any swallowing difficulties and ensure airway protection (Hirano). Domperidone can be used for gastrointestinal symptoms of nausea and vomiting in mitochondrial disease patients. Gastrostomy and parenteral feeding may become necessary for nutritional support (Hirano). Amitriptyline, nortriptyline, and gabapentin can be used for neuropathic symptoms (Hirano). The neurological system is often involved in patients with mitochondrial disease due to the high energy demands of the cells in the nervous system. Some patients may require specialized schooling arrangements including schooling at home as they are unable to attend school due to physical or other challenges. As an example, in Mitochondrial Neurogastrointestinal Encephalopathy (MNGIE) disease feeding tubes and/or parenteral nutrition such as Total Parenteral Nutrition (TPN) are required or utilized (Hirano).

## Conclusion

Mitochondrial diseases are caused by genetic mutations in either mtDNA or nuclear DNA and can cause a diverse range of symptoms affecting virtually all organ systems. At this time there is no cure for these diseases. While there are some promising experimental approaches in mitochondrial research, moving from preclinical research into clinical approaches is proving to be challenging because mitochondrial diseases are very complex. Further collaboration between basic research laboratories and industry is required to overcome challenges.

## Additional Information and Declarations

### Data Availability

The following information was supplied regarding data availability: The research in this article did not generate any data or code; this manuscript is a literature review.
